# The impact of viewing a video with and without head phones on snack intake: A pilot study

**DOI:** 10.1371/journal.pone.0188457

**Published:** 2017-12-07

**Authors:** Anastasia Dieze, Theodora Stephan, Carolin Hilzendegen, Nanette Stroebele-Benschop

**Affiliations:** Institute of Nutritional Medicine, Department of Nutritional Psychology, University of Hohenheim, Stuttgart, Germany; University of Oslo, NORWAY

## Abstract

Research shows that many small changes to the environment impact one’s eating behavior. The aim of this study was to examine whether the type of audio transmission would affect snack intake depending on the degree of immersion. A sample of 174 university students were randomized to either viewing a movie wearing headphones or listening over loud speakers while consuming a snack of their choice. Significant differences were found with more snacks consumed in the group without headphones compared to the group wearing headphones. Particularly women tend to eat less (about 10% of the offered snack less) when wearing headphones while viewing a movie. The results seem to indicate that audio transmission mode might impact eating behavior.

## Introduction

Given the continuous rise of overweight and obesity prevalence globally, scientists have started to study the impact of the eating environment on food intake more intensively [1,2]. Besides individually based treatment and prevention approaches, research in this area is looking for possibilities to change the eating environment to reduce intake and promote healthy eating [3–5].

Various studies have, for instance, examined the influence of television viewing and media use on food intake [6, 7] and experimental studies have tested the impact of distraction via music or television viewing while consuming a meal [8–10].

Listening to music, watching television or playing video games while eating appear to be distracting activities associated with increased food intake [11–13]. Intervention studies have often included the reduction of activities such as television viewing or video game playing as approaches to reduce weight or prevent weight gain in children and adults [14–16]. While these activities are considered sedentary activities and therefore not recommended for a healthy active lifestyle, they also seem to be adding additional calories to the overall food intake by increased total intake [14, 17], increased snacking frequency [18], and by a greater exposure to marketing of energy dense foods which can lead to increased intake of advertised foods [19, 20].

The level of distraction during food intake can be explained with the level of immersion. Immersion is described as a state of being deeply engaged or absorbed in a certain activity. The more a person is immersed into an activity or situation, the less s/he is aware of his/her surroundings [21–23]. While viewing television, for example, one’s attention is turned to the television program at the expense of one’s surroundings. Listening to the radio or music also seems to distract from the act of eating. However, it appears to do so less than viewing television [11, 12, 24]. Hetherington and colleagues revealed that study participants only looked at their plate when the television was on during 28% of the mealtime compared to 85% when eating alone and without viewing television [25].

In fact, different forms of media appear to have different immersion tendencies [16]. Narrative-based media seem to be more distracting and thus leading to greater energy intake than non-narrative media [12, 26]. Interestingly, one study found no differences in immersion variables comparing video games and television watching except when looking at the immersion into the storyline also described as “narrative transportation”. Narrative transportation was higher in the TV group than the gaming group and it was also significantly associated with total caloric intake [26]. The authors concluded that some forms of distraction such as the storyline may affect eating behavior while others do not impact caloric intake.

The aim of this pilot study was to examine whether the distraction while viewing television by increasing the level of immersion via headphones would impact snack intake. According to Kallinen and Ravaja (2007), the disengagement from the surroundings while viewing television can be increased by using headphones [22]. It was hypothesized that by using headphones to increase the level of immersion, snack intake during a television show would be increased compared to television viewing via loud speakers.

## Materials and methods

### Subjects

The participants were university students recruited via posted advertisements, flyers, a social networking site on Facebook and the university’s online newsletter. Criteria for inclusion were being a student aged between 18–30 years and speaking German. Exclusion criteria were food intolerances or allergies towards the offered snacks or studying nutritional sciences in order to avoid bias caused by the potential knowledge regarding mindful eating behavior. Informed consent was obtained on the study day, before starting the experiment. The participants were entered in a raffle to win a semester train ticket as an incentive for their participation. The present study was conducted according to the guidelines of the Declaration of Helsinki. All procedures were approved by the local Ethics Committee of the University of Hohenheim, Germany.

### Experimental design

To examine the effect of audio transmission type on consumed snack amount, a randomized experimental study was conducted from November 2016 for 4 months. Using a computer-based pseudo-random number generator, subjects were able to register online for different days, which were blockwise randomized in a one to one ratio. A power analysis indicated that a total sample of 182 participants would be needed in order to detect a difference of 12% of consumed snacks between the two groups (with 80% power and alpha at .05), basing the calculation on results of a study by Peneau et al. [10].

Subjects were invited to watch a 40-minute movie after being randomized into two groups differing only in the type of audio transmission, by either listening to the movie via speakers (SP group) or via headphones (HP group). Headphones have been repeatedly used in sensory research, e.g. when investigating the impact of noise and sounds on taste perception [27].

Before their scheduled appointment, subjects were asked via email or phone which of the following snacks they would like to consume during the movie. The snack choices included two salty snacks (potato chips and salted peanuts) and two sweet snacks (chocolate coated peanuts and cola flavored gummy bears) ranging from 130 grams (for potato chips) to 400 grams (for cola flavored gummy bears). The calories and energy content of each snack (100g) are presented in S1 Table available online. The snacks were offered in bowls (ranging in diameter from 17cm to 23 cm) to be consumed ad libitum.

The movie shown was the first episode of a British crime drama television season called ‘*Happy Valley*’ (Sally Wainwright, Red Productions/BBC, 2015). The episode was considered exciting for both gender and was generally unknown to the German population.

### Procedures

Students were seated in one of six comfortable lounge chairs. Before the start of the movie they were asked to complete a demographic questionnaire including age, gender, self-reported height and weight. The German version of the Dutch Eating Behavior Questionnaire (DEBQ) was used to measure eating styles [28, 29]. The DEBQ measures three eating styles (emotional eating, external eating, restraint) and comprises 33 items responded to on a Likert-scale from 1 = *seldom* to 5 = very often. The questionnaire was chosen to control for possible food-related behavioral problems.

Furthermore, the participants estimated their subjective hunger and appetite state on a Visual Analogue Scale from 1 (not hungry/no appetite) to 10 (very hungry/much appetite) as well as the last time they ate before the experiment. They were then instructed to watch the movie while snacking. Participants were told not to share their snacks with other participants nor to talk during the movie. The sound samples were calibrated using a WS13161C digital sound level meter (Wensn, China). The average loudness was 57.9 (± 6.9) dB. After watching the movie, participants answered another questionnaire which included questions about the subject’s comfort, liking of the snack and movie (7-Point-Likert scales from 1—extremely bad to 7—extremely good) as well as ratings of immersion. Immersion was measured by five items based on the questionnaire from *Oh et al*. including fascination, absorption, focus, mental involvement and emotional affection (Likert scales 1—not at all applicable to 7—very applicable) as representing different depths of immersion [30].

The movie was rated regarding the general impression, tension and boredom on 7-Point-Likert scales, respectively. The amount and caloric content of the snacks eaten were recorded by the experimenter by weighing the served bowls before and after consumption.

### Statistical analyses

Main outcome of the study was the proportion of consumed snacks depending on whether participants watched the movie via headphones or speakers. Baseline characteristics, eating styles, liking of the movie and variables about immersion of the two test groups were analyzed by Mann-Whitney U and Pearson’s χ^2^-tests. Due to the right-skewed distribution Mann-Whitney U tests were conducted to compare the amount of consumed snacks of HP and SP group, also when adjusting for gender. Finally, a Spearman correlation was calculated to examine the relationship between immersion and consumed snacks. Statistical significance level was set at *p* < 0.05. Statistical tests were carried out using IBM SPSS Statistics for Windows, version 23.0.

## Results

A total of 177 students participated in the study. Three participants were excluded, one due to the lack of informed consent, one who was older than 30 years and one because of technical problems with the headphones. Therefore, the results of 174 participants (64 men and 110 women) were analyzed. Mean age was 22.09 (±2.79) years (range: 18–30 years) and the mean BMI was 22.67 (±3.44) kg/m^2^ (range: 16.85–37.50 kg/m^2^). The type of snack chosen by the subjects are shown in Table 1. No significant differences between the two test groups were found in terms of age, gender, BMI, eating styles, hunger, appetite, time since the last meal, movie liking, and chosen snack type, as shown in Table 1.

**Table 1:** Participant characteristics.

	Total sample (n = 174)	Test group		
		Speakers (n = 89)	Headphones (n = 85)	
Age (in years)	22.09 (2.79)	22.02 (2.54)	22.31 (3.37)	*Z* = -0.067, n.s.
Gender				
Male	36.8	39.3	34.1	*Χ*^*2*^ = 0.592, n.s.
Female	63.2	60.7	65.9	
BMI (kg/m^2^)	22.67 (3.44)	22.73 (3.76)	22.60 (3.08)	*Z* = -0.376, n.s.
Snack type				
Potato Chips	36.2	38.2	34.1	
Salted peanuts	19.5	18.0	21.2	
Chocolate coated peanuts	27.0	29.2	24.7	*Χ*^*2*^ = 1.382, n.s.
Cola flavored gummy bears	17.2	14.6	20.0	
Hunger	4.21 (2.46)	4.21 (2.58)	4.22 (2.34)	*Z* = -0.086, n.s.
Appetite	6.17 (1.96)	6.19 (1.94)	6.15 (1.99)	*Z* = -0.093, n.s.
Minutes since last meal	203.52 (131.60)	197.98 (103.06)	209.33 (156.45)	*Z* = -0.113, n.s.
Restraint	2.42 (0.80)	2.39 (0.86)	2.46 (0.75)	*Z* = -0.657, n.s.
Emotional Eating	2.26 (0.67)	2.26 (0.69)	2.27 (0.66)	*Z* = -0.182, n.s.
External Eating	3.45 (0.42)	3.48 (0.41)	3.42 (0.43)	*Z* = -1.095, n.s.
Movie liking	5	5	5	*Z* = -0.271, n.s.
Immersion	4.34 (1.12)	4.31 (1.22)	4.38 (1.01)	*Z* = -0.258, n.s.
Fascination	4	4	4	*Z* = -0.382, n.s.
Absorption	5	5	5	*Z* = -0.626, n.s.
Focus	5	5	5	*Z* = -0.410, n.s.
Mental involvement	5	5	5	*Z* = -0.660, n.s.
Emotional affection	4	4	4	*Z* = -0.528, n.s.

*Note*. Gender and snack type are presented in percent, age, BMI, eating styles, hunger, appetite, minutes since last meal and immersion as mean (*SD*). Other variables are presented as median of a 7-point-Likert scale.

### Differences in snack consumption

**Fig 1:**
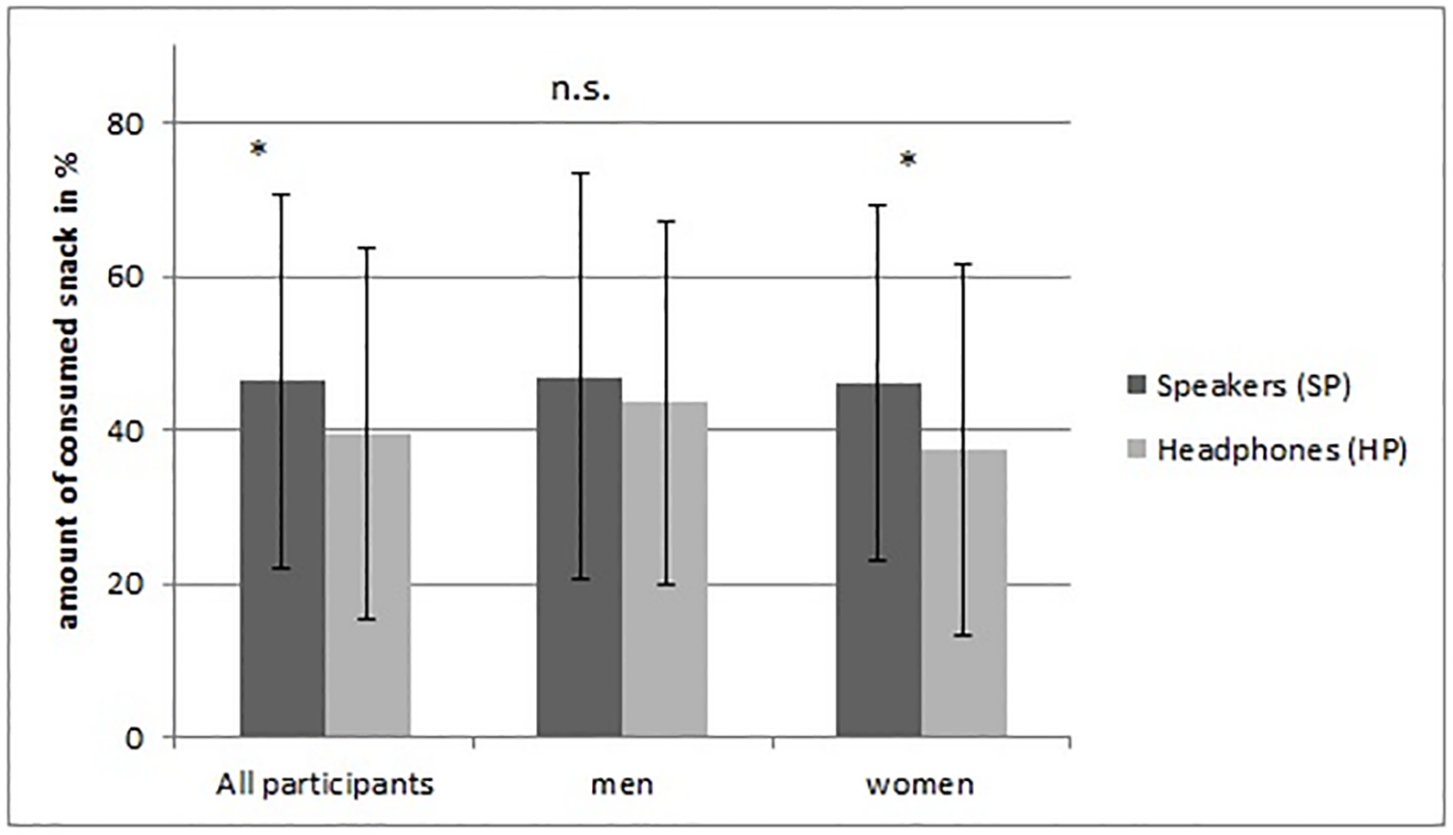
Differences in snacks consumed (in mean percentage, SD) across the SP and HP groups for the total sample and separated by gender.

The proportion of snacks consumed differed significantly between the two test groups (Fig 1). However, the participants in the SP group ate more of the served snacks (46.45% ± 24.34) than the participants in the HP group (39.53% ± 24.12, *Z* = --2.22; *p* = 0.026).

Mann-Whitney U Tests adjusted for gender showed different results for men and women. For men only, the difference between the SP and the HP group was no longer significant (SP: 46.86% ± 26.38 vs. HP: 43.54% ± 23.75, *Z* = -0.48, n.s.) while the effect was even more pronounced for women (SP: 46.18% ± 23.17 vs. HP: 37.46% ± 24.25, *Z* = -2.33, *p* = 0.020). Testing for significant differences in calories or grams, the same albeit not significant tendencies could be detected (calories: SP group: 500.39 kcal ± 284.56 vs. HP group: 430.53 kcal ± 275.66, *Z* = -1.84, *p* = 0.066; grams: SP group: 106.08 grams ± 62.95 vs. HP group: 90.76 grams ± 60.15, *Z* = -1.90, *p* = 0.058).

### Differences in immersion ratings

Overall, the participants indicated to rather like the movie (4.48 ± 1.27, Mdn = 5, Range = 1–7). There were no differences between the two groups. The majority of the participants rated the movie as captivating and not boring (Captivating: 4.71 ± 1.29, Mdn = 5, Range = 1–7, Boring (reverse scored): 4.92 ± 1.35, Mdn = 5, Range = 1–7). No differences between the test groups in terms of movie liking and no gender differences in immersion ratings were found.

Combining all five immersion variables into one overall immersion score to calculate overall immersion and comparing both test groups revealed no significant differences between the mean immersion ratings across the test groups (SP: 4.31 ± 1.22, HP: 4.38 ± 1.02; *Z* = -0.26, n.s.). When looking at the immersion variables separately also revealed that none of the single variables showed significant differences between the test groups. Using a Spearman correlation showed no relationship between the overall immersion variable and the percentage of snacks consumed (*r*_*S*_ = -0.088, n.s.).

## Discussion

Contrary to our hypothesis that wearing headphones while viewing a movie would lead to higher snack intake compared to viewing a movie over loud speakers, our results showed that participants ate significantly more snacks when viewing a movie without wearing headphones.

Most importantly, it needs to be stated that participants, contrary to our assumption, did not seem to be more immersed in the movie when wearing headphones. Immersion ratings did not differ between the two groups. It is unclear whether wearing headphones did not increase immersion while viewing a movie or whether the used questionnaire failed to adequately assess immersion in this study.

Nevertheless, the results, especially for the female sample, showed significantly different results between using headphones and speakers. For instance, compared to the headphone group, female participants in the speakerphone group consumed on average about 23% more (46.18% vs. 37.46% of the offered snack). Depending on the type of snack consumed, e.g. for chocolate coated peanuts, this would be equivalent to 100 calories less when using headphones while viewing a movie. The caloric difference is minor but considering the small changes approach [31, 32] to prevent weight gain on a population-based level, such a small daily difference in caloric intake could make a difference in body weight in the long term.

Potential mechanisms for this effect can only be speculative in nature. While using headphones seems common in transit or while listening to music, it might still be a distracting and unfamiliar circumstance while viewing television. The salience of this condition could have negatively affected intake.

When looking at the gender differences, it is possible that the differences in eating styles play a role in the results. Research has shown that women tend to have higher scores on restraint and emotional eating style scales [33, 34] and that distraction induced food intake might depend on the level of dietary restraint with higher levels of restraint being associated with higher intake [35]. In our study, female participants also showed significantly higher scores on the emotional eating and restrained eating scale compared to the male participants but no association between immersion and eating styles were found. Thus, it needs to be further investigated whether headphones cause people to be less distracted while viewing a movie and whether this could cause people with higher restraint scores to eat less compared to eating while viewing a movie without headphones.

## Conclusion

Thus, while these finding are very interesting, further investigating of this new unexplored potential environmental influence on food intake is necessary. Since a sensible explanation for these findings including the existing gender difference seems to be missing and given the importance of reducing snacking while viewing a movie or watching television to avoid potential weight gain, the mechanisms that play a role in these differences in snack intake need to be further evaluated and examined. Studies examining possible underlying mechanisms and confirming our results are needed before making any recommendations regarding people’s eating behavior. Future studies should add different measures of immersion and distraction, use different genres of movies and use a real-life approach by having participants watch television with and without headphone in their own home.

## Supporting information

S1 Table (DOCX)
