# Supplementary material for: The impact of viewing a video with and without head phones on snack intake: A pilot study
Source: PLoS One. 2017 Dec 7;12(12):e0188457. doi: 10.1371/journal.pone.0188457 (PMC5720623; doi:10.1371/journal.pone.0188457)
Supplement: S1 Table — (DOCX) [file pone.0188457.s001.docx]

S1 Table

S1 Table. Calories per 100g and macronutrient composition of offered snack types

|  |  | Macronutrients by 100g [g] | | | |
| --- | --- | --- | --- | --- | --- |
|  | kcal by 100g | Fat | Carbohydrates | Sugar | Protein |
| Potato Chips | 529 | 33 | 49 | 2.0 | 5.8 |
| Salted peanuts | 620 | 51 | 9.6 | 5.2 | 27 |
| Chocolate coated peanuts | 500 | 25 | 61 | 58 | 7.8 |
| Cola flavored gummy bears | 343 | 0.5 | 77 | 46 | 6.9 |
